# Effects of physical exercise on negative emotions in adolescents: Sequential mediation through psychological benefits and social self-efficacy

**DOI:** 10.1016/j.pmedr.2025.103231

**Published:** 2025-08-29

**Authors:** Xin Yang, Liuheng Lu

**Affiliations:** a Guangxi University, Nanning 530004, China; b Guangxi Orthopedic Hospital, Nanning 530012, China

**Keywords:** Adolescent development, Emotional regulation, Physical exercise, Psychological benefits, Social self-efficacy, Exercise psychology

## Abstract

**Objective:**

Negative emotions during adolescence constitute a significant public health challenge requiring theoretically-grounded intervention approaches. This investigation examined sequential mediation mechanisms whereby physical exercise influences adolescent negative emotions through psychological benefits and social self-efficacy pathways, integrating neurobiological and social-cognitive theoretical frameworks.

**Methods:**

Cross-sectional analysis of 1471 Chinese adolescents (Mean age = 13.16 years) from Guangxi, China, with data collected in September 2024, employed structural equation modeling with bias-corrected bootstrapping to test hypothesized sequential mediation pathways. Physical exercise, psychological benefits, social self-efficacy, and negative emotions were assessed using validated instruments.

**Results:**

The sequential mediation model showed acceptable fit (χ^2^/df = 3.09, comparative fit index = 0.97, root mean square error of approximation = 0.04, standardized root mean square residual = 0.04). Physical exercise was directly associated with lower negative emotions and indirectly through psychological benefits. A sequential pathway from exercise → psychological benefits → social self-efficacy → negative emotions was also observed. The model explained 56 % of the variance in negative emotions.

**Conclusions:**

Findings demonstrate that exercise influences adolescent negative emotions through both direct neurobiological mechanisms (31.7 % of total effect) and sequential psychological adaptation processes (68.3 % of total effect). Programs that enhance perceived psychological gains from exercise and build social efficacy may deliver meaningful emotional benefits.

## Highlights

• Physical exercise influences adolescent emotions through psychological pathways.
• Psychological benefits serve as primary mediator in exercise-emotion relationships.
• Social self-efficacy provides additional therapeutic value through sequential pathways.
• Sequential mediation reveals cascading psychological adaptation in adolescent exercise.

## 1 Introduction

Negative emotions during adolescence constitute a significant public health challenge, with approximately 32 % of adolescents experiencing clinically significant emotional distress during this critical developmental period (Mesurado et al., 2018; Rodriguez-Ayllon et al., 2019; Reitsema et al., 2022; Poon et al., 2022). This prevalence coincides with neurobiological vulnerabilities characterized by differential maturation of prefrontal regulatory regions relative to limbic emotion-generating systems, creating heightened emotional reactivity coupled with developing regulatory capacity (Casey et al., 2008; Zschucke et al., 2015; Poon et al., 2022).

### 1.1 Neurobiological mechanisms of exercise-emotion relationships

Physical exercise has emerged as a promising intervention approach for addressing adolescent negative emotions, operating through documented neurobiological pathways (Wang et al., 2024). Enhanced brain-derived neurotrophic factor (BDNF) expression supports neuroplasticity in emotion-regulatory circuits, particularly strengthening connectivity between prefrontal regulatory regions and limbic emotional-processing systems (Lin and Kuo, 2013; Rodriguez-Ayllon et al., 2019; Sun et al., 2023). Exercise modulates hypothalamic-pituitary-adrenal axis functioning, reducing stress reactivity and cortisol dysregulation commonly observed in adolescent emotional disorders (Zschucke et al., 2015; Sun et al., 2023). Additionally, altered monoaminergic neurotransmission in serotonergic and dopaminergic systems contributes to mood stabilization and enhanced emotional resilience (Lin and Kuo, 2013; Gu et al., 2024). Neuroimaging studies demonstrate exercise-induced modulation of neural circuits implicated in emotional regulation during adolescence (Hwang et al., 2023), with the adolescent brain's heightened neuroplasticity creating critical windows wherein exercise interventions may yield particularly pronounced benefits (Herting and Chu, 2017; Sun et al., 2023).

### 1.2 Social-cognitive developmental mechanisms

Social Cognitive Theory (Bandura, 2001) provides complementary understanding of how physical exercise enhances psychological functioning through social-cognitive pathways. Mastery experiences acquired through successful participation in physical activities contribute to enhanced self-efficacy beliefs, potentially transferring across domains through generalized competence perceptions. The social context of physical activities provides structured opportunities for developing social skills, receiving performance feedback, and experiencing social success, thereby enhancing social self-efficacy beliefs (McPhie and Rawana, 2015; Wu et al., 2025). Social self-efficacy—an individual's perceived capability to engage effectively in social interactions and establish interpersonal relationships—represents critical developmental capacity during adolescence, when peer relationships gain increasing importance and social cognitive capacities undergo significant maturation (Blakemore and Mills, 2014). Robust negative associations between social self-efficacy and emotional distress indicators have been empirically established, with effect sizes commonly exceeding Cohen's medium threshold (Smith and Betz, 2000; Peng et al., 2025).

### 1.3 Sequential mediation theoretical framework

To address theoretical limitations in previous unidimensional models, we propose a sequential mediation framework integrating neurobiological and social-cognitive mechanisms within developmental contexts. This integration advances beyond simple direct-effect models by recognizing cascading exercise-induced psychological adaptations during adolescence. The Broaden-and-Build Theory (Fredrickson, 2001) provides theoretical foundation for understanding how psychological benefits derived from physical exercise initiate positive developmental cascades. Positive experiences including enjoyment and accomplishment during physical activity broaden cognitive-behavioral repertoires and build enduring psychological resources enhancing emotional regulation capacity. These psychological benefits create upward spirals of engagement, setting the stage for subsequent social-cognitive development with cumulative effects exceeding simple additive models.

The proposed sequential mediation model posits that physical exercise first enhances psychological benefit perceptions, subsequently facilitating social self-efficacy development, ultimately contributing to substantial reductions in negative emotions (Yuan et al., 2022). This temporal sequencing aligns with developmental principles wherein affective experiences precede and shape cognitive-behavioral adaptations. Sequential processes may be particularly pronounced during adolescence due to developmental peaks in reward sensitivity and dopaminergic activity (Galván, 2013), potentially amplifying cascading effects of subjective rewards on psychological development.

### 1.4 Study hypotheses

Based on this integrated theoretical framework, we propose:

Hypothesis 1 Physical exercise will demonstrate significant negative relationships with negative emotions in adolescents.

Hypothesis 2 Psychological benefits will substantially mediate the exercise-emotion relationship.

Hypothesis 3 Social self-efficacy will mediate the relationship between psychological benefits and negative emotions.

Hypothesis 4 Sequential mediation will emerge whereby exercise influences negative emotions through psychological benefits, which influences emotions through social self-efficacy, with combined indirect effects accounting for the majority of the total relationship.

## 2 Methods

### Study design and participants

This cross-sectional investigation, conducted in September 2024, employed multi-stage stratified cluster sampling to examine exercise–emotion regulatory pathways through developmental perspectives. Ten middle schools in Guangxi Province, China were selected with stratification across urban (*n* = 4) and rural (*n* = 6) environments, controlling for urbanicity-associated confounds in physical activity resource accessibility (Rodriguez-Ayllon et al., 2019). The analytical sample comprised 1471 adolescents (effective response rate: 98.07 %; 802 males, 669 females; mean age = 13.16 years, SD = 1.01), with balanced grade distribution (grade 7: 33.92 %; grade 8: 33.45 %; grade 9: 32.63 %). Demographic comparison with provincial census data demonstrated non-significant differences in gender distribution (χ^2^ = 2.31, *p* = 0.13) and urban-rural proportion (χ^2^ = 1.89, *p* = 0.17), supporting sample representativeness.

Comprehensive institutional ethics committee approval and multi-level consent procedures (parental consent/adolescent assent) were implemented following established protocols for developmentally-sensitive research with adolescent populations. Inclusion criteria required: (1) current enrollment in participating schools, (2) absence of diagnosed physical limitations preventing exercise participation, and (3) completion of all study measures. Exclusion criteria included: (1) chronic medical conditions affecting exercise capacity, and (2) current participation in structured psychological interventions.

## 3 Measures

### 3.1 Physical exercise

Physical exercise was operationalized as structured bodily movement requiring energy expenditure above resting levels, deliberately performed for health, fitness, or recreational purposes, distinguishing intentional exercise from incidental physical activity (Ekkekakis and Brand, 2019). The Youth Physical Exercise Rating Scale (Liang, 1994) assessed exercise participation across intensity, frequency, and duration dimensions through 8 items using 5-point Likert scaling. Exercise volume was calculated as: Frequency × (Time-1) × Intensity. The scale demonstrated excellent internal consistency (Cronbach's α = 0.85) and satisfactory confirmatory factor analysis fit (comparative fit index (CFI) = 0.93; root mean square error of approximation (RMSEA) = 0.04).

### 3.2 Psychological benefits

Psychological benefits were conceptualized as perceived positive psychological outcomes derived from physical exercise participation, addressing cognitive, social, and affective dimensions of exercise benefits. This construct was assessed using an adapted Psychological Benefits Scale (Liu, 2017), evaluating perceived personality expression, social recognition, and enjoyment derived from physical activity through 3 items. The scale demonstrated excellent internal consistency (Cronbach's α = 0.89) and strong model fit (CFI = 0.93; RMSEA = 0.04). This operationalization aligns with Broaden-and-Build theoretical frameworks emphasizing subjective benefit perception as catalysts for developmental processes.

### 3.3 Social self-efficacy

Social self-efficacy was operationalized as perceived capability to engage effectively in social interactions and establish interpersonal relationships. The Social Self-Efficacy Scale (Smith and Betz, 2000) assessed this construct through 18 items measuring confidence in social situations and interpersonal tasks using 5-point Likert scaling ranging from 1 (no confidence) to 5 (complete confidence). Strong internal consistency (Cronbach's α = 0.86) and satisfactory confirmatory factor analysis fit (CFI = 0.93; RMSEA = 0.05) were demonstrated. This instrument's selection reflects Social Cognitive Theory principles emphasizing self-efficacy's role in adolescent social development.

### 3.4 Negative emotions

Negative emotions were conceptualized as aversive affective states characterized by subjective distress and unpleasant valence, encompassing anxiety, sadness, anger, and shame dimensions. The 10-item Negative Affect Scale from the Positive and Negative Affect Schedule (Watson et al., 1988) measured emotional experience frequency using 5-point Likert scaling from 1 (very slightly) to 5 (extremely). Strong internal consistency (Cronbach's α = 0.83) and acceptable model fit (CFI = 0.92; RMSEA = 0.05) were observed.

### 3.5 Data collection and analysis

Data collection occurred during regular school hours using standardized protocols with trained research assistants. Methodological safeguards included counterbalanced instrument ordering, physical separation between participants, and neutral instruction phraseology to minimize bias. Model fit evaluation employed multiple indices: χ^2^/df ratio < 3.0, CFI ≥ 0.95, RMSEA ≤0.06, and standardized root mean square residual ≤0.08. Sequential mediation effects were examined through indirect effect decomposition, with significance determined by bias-corrected 95 % confidence intervals excluding zero. Complex sampling effects were acknowledged as potential limitations requiring future multilevel modeling approaches. The hypothesized sequential mediation model was tested using structural equation modeling in AMOS 24.0 with maximum likelihood estimation and bias-corrected bootstrapping (5000 resamples).

## 4 Results

### 4.1 Preliminary analyses and descriptive statistics

Common method variance assessment through Harman's single-factor test yielded four factors with eigenvalues exceeding unity, with the primary factor accounting for 22.84 % of total variance—below the 40 % threshold, suggesting minimal method bias. Multicollinearity diagnostics confirmed parameter estimation stability (variance inflation factor range: 1.12–1.89). Descriptive statistics and correlations are presented in Table 1. Physical exercise demonstrated substantial negative correlation with negative emotions (*r* = −0.41, *p* < 0.01), robust positive associations with psychological benefits (*r* = 0.52, *p* < 0.01) and social self-efficacy (*r* = 0.44, p < 0.01). Psychological benefits showed strong correlations with social self-efficacy (*r* = 0.56, *p* < 0.01) and negative emotions (*r* = −0.63, p < 0.01). Social self-efficacy exhibited robust negative correlation with negative emotions (*r* = −0.47, p < 0.01), providing strong initial support for the sequential mediation framework.

**Table 1:** Descriptive statistics and bivariate correlations of key variables among Chinese adolescents *(Guangxi, China; September 2024; n = 1471).*

Variables	*M*	*SD*	1	2	3	4
1. Physical Exercise	2.73	0.72	1			
2. Psychological Benefits	1.64	0.66	0.52**	1		
3. Social Self-efficacy	2.31	0.62	0.44**	0.56**	1	
4. Negative Emotions	1.92	0.93	−0.41**	−0.63**	−0.47**	1

***Note.*** Measurement details: Physical Exercise was assessed using the Youth Physical Exercise Rating Scale (Liang, 1994; 8 items; 5-point Likert); exercise volume was computed as frequency × (time − 1) × intensity; higher scores indicate greater participation (Cronbach's α = 0.85). Psychological Benefits were measured with an adapted Psychological Benefits Scale (Liu, 2017; 3 items covering enjoyment, personality expression, and social recognition; 5-point Likert; α = 0.89). Social Self-efficacy was measured using the Social Self-Efficacy Scale (Smith and Betz, 2000; 18 items; response options 1–5; α = 0.86). Negative Emotions were measured by the 10-item Negative Affect subscale of the PANAS (Watson et al., 1988; response options 1–5; α = 0.83). Correlations are two-tailed; **p < 0.01.

### 4.2 Sequential mediation model results

The structural equation model demonstrated excellent fit: χ^2^ = 142.18, df = 46, p < 0.01; χ^2^/df = 3.09; comparative fit index = 0.97; root mean square error of approximation = 0.04 (90 % CI [0.03, 0.05]); standardized root mean square residual = 0.04. Path coefficients and their significance levels are presented in Table 2.

**Table 2:** Standardized path coefficients of the sequential mediation model linking physical exercise to negative emotions via psychological benefits and social self-efficacy *(Chinese adolescents; Guangxi, China; September 2024; n = 1471).*

Path	β	SE	Critical Ratio	95 % CI
Physical Exercise →Psychological Benefits	0.52	0.04	13.00	[0.44, 0.60]
Psychological Benefits →Social Self-Efficacy	0.56	0.04	14.00	[0.48, 0.64]
Physical Exercise →Negative Emotions (direct)	−0.16	0.04	−4.00	[−0.24, −0.08]
Psychological Benefits →Negative Emotions	−0.41	0.04	−10.25	[−0.49, −0.33]
Social Self-Efficacy →Negative Emotions	−0.19	0.04	−4.75	[−0.27, −0.11]

***Note.*** Measurement details: Physical Exercise—Youth Physical Exercise Rating Scale (Liang, 1994; 8 items; 5-point Likert; exercise volume = frequency × (time − 1) × intensity; α = 0.85). Psychological Benefits—adapted Psychological Benefits Scale (Liu, 2017; 3 items; 5-point Likert; α = 0.89). Social Self-efficacy—Social Self-Efficacy Scale (Smith and Betz, 2000; 18 items; 1–5; α = 0.86). Negative Emotions—PANAS Negative Affect (Watson et al., 1988; 10 items; 1–5; α = 0.83). SE = standard error. Critical Ratio = estimate / SE. 95 % CI = bias-corrected 95 % confidence interval from 5000 bootstrap resamples.

Physical exercise significantly predicted psychological benefits (β = 0.52, p < 0.01), representing a large effect confirming the first sequential link. Psychological benefits significantly predicted social self-efficacy (β = 0.56, p < 0.01), establishing robust second sequential linkage. Both psychological benefits (β = −0.41, p < 0.01) and social self-efficacy (β = −0.19, p < 0.01) significantly predicted reduced negative emotions. The direct effect of physical exercise on negative emotions remained significant (β = −0.16, p < 0.01), indicating partial mediation through proposed psychological mechanisms.

Sequential mediation analysis revealed multiple significant pathways with clinically meaningful magnitudes (Table 3). Simple mediation through psychological benefits was substantial (β = −0.21, 95 % CI [−0.27, −0.16]), strongly supporting Hypothesis 2. The sequential mediation pathway—exercise through psychological benefits, then through social self-efficacy to negative emotions—demonstrated significant effects (β = −0.06, 95 % CI [−0.09, −0.03]), supporting Hypothesis 4. The total indirect effect (β = −0.28, 95 % CI [−0.34, −0.22]) accounted for 68.3 % of the total effect, while direct effects retained substantial magnitude (β = −0.13, 31.7 % of total effect), confirming partial mediation. The model explained 27 % of variance in psychological benefits, 31 % in social self-efficacy, and 56 % in negative emotions.

**Table 3:** Bias-corrected bootstrapped indirect effects (95 % confidence intervals) for the sequential mediation model *(Chinese adolescents; Guangxi, China; September 2024; n = 1471).*

Indirect Path	Effect	Boot SE	95 % CI	% of Total Effect
Physical Exercise →Psychological Benefits →Negative Emotions	−0.21	0.03	[−0.27, −0.16]	51.2 %
Physical Exercise →Social Self-Efficacy →Negative Emotions	−0.01	0.02	[−0.04, 0.02]	2.4 %
Physical Exercise →Psychological Benefits →Social Self-Efficacy →Negative Emotions	−0.06	0.02	[−0.09, −0.03]	14.6 %
Total Indirect	−0.28	0.03	[−0.34, −0.22]	68.3 %
Direct Effect	−0.13	0.04	[−0.21, −0.05]	31.7 %
Total Effect	−0.41	0.04	[−0.49, −0.33]	100.0 %

***Note.*** Indirect paths and effects are standardized. Boot SE = bootstrap standard error; 95 % CI = bias-corrected 95 % confidence interval based on 5000 resamples. Measurement details: Physical Exercise—Youth Physical Exercise Rating Scale (Liang, 1994; 8 items; 5-point Likert; exercise volume = frequency × (time − 1) × intensity; α = 0.85). Psychological Benefits—adapted Psychological Benefits Scale (Liu, 2017; 3 items; 5-point Likert; α = 0.89). Social Self-efficacy—Social Self-Efficacy Scale (Smith and Betz, 2000; 18 items; 1–5; α = 0.86). Negative Emotions—PANAS Negative Affect (Watson et al., 1988; 10 items; 1–5; α = 0.83).

## 5 Discussion

This investigation provides empirical evidence for sophisticated sequential mediation mechanisms explaining exercise-emotion relationships in adolescence, wherein physical exercise influences negative emotions through both direct neurobiological pathways and indirect psychological adaptation processes. The partial mediation pattern—with 68.3 % of total effects operating through psychological pathways while 31.7 % represents direct exercise-emotion effects—offers nuanced theoretical insights with substantial clinical implications for adolescent mental health intervention development.

### 5.1 Sequential mediation mechanism interpretation

The substantial indirect effect through psychological benefits (β = −0.21, representing 51.2 % of total effect) establishes this mechanism as the predominant pathway through which exercise influences emotional regulation in adolescence (Li et al., 2024). This finding extends Broaden-and-Build Theory (Fredrickson, 2001) by demonstrating that exercise-induced positive experiences serve as primary catalysts for emotional benefits, with effect sizes exceeding those typically observed in intervention studies (Wang et al., 2024). The magnitude suggests that adolescents' subjective interpretations of exercise experiences—encompassing enjoyment, personal accomplishment, and social recognition—constitute the most potent mechanism for emotional regulation enhancement. From developmental perspectives, this aligns with heightened reward sensitivity characteristic of adolescent neurodevelopment (Galván, 2013), where large effect sizes (β = 0.52) for exercise-psychological benefits relationships indicate particularly strong responsiveness to rewarding aspects of physical activity.

The significant sequential mediation pathway (β = −0.06, representing 14.6 % of total effect) provides novel evidence for cascading psychological adaptation processes whereby initial exercise-induced positive experiences facilitate subsequent social competence development. This extends Social Cognitive Theory (Bandura, 2001) by demonstrating temporal sequencing in self-efficacy development, wherein affective experiences precede cognitive-behavioral adaptations (Peng et al., 2025). The robust relationship between psychological benefits and social self-efficacy (β = 0.56) suggests positive exercise experiences provide foundational conditions for enhanced social confidence through mastery experience generalization. During adolescence, when social brain networks undergo substantial reorganization (Blakemore and Mills, 2014), exercise-induced competence experiences may transfer more readily to social domains than in other developmental periods (Christner et al., 2024).

The retained direct effect (β = −0.16, representing 31.7 % of total effect) indicates exercise influences emotional regulation through mechanisms beyond measured psychological pathways, likely reflecting neurobiological processes operating independently of conscious psychological adaptation. This supports integration of neurobiological perspectives with social-cognitive frameworks, suggesting exercise benefits emerge through multiple, complementary mechanisms. The direct effect likely encompasses exercise-induced neuroplastic adaptations, including enhanced BDNF expression, modified neurotransmitter functioning, and altered prefrontal-limbic connectivity patterns (Lin and Kuo, 2013; Herting and Chu, 2017), representing automatic regulatory mechanisms developing through repeated exercise exposure.

### 5.2 Clinical significance and effect size implications

The observed effect sizes demonstrate substantial clinical significance for adolescent mental health applications. The total effect (β = −0.41) represents large effects according to Cohen's conventions and substantially exceeds meta-analytic findings for exercise interventions in youth populations (Rodriguez-Ayllon et al., 2019). The 56 % variance explained in negative emotions approaches levels observed in established psychotherapeutic interventions, indicating meaningful potential for exercise-based approaches in clinical and preventive contexts. The partial mediation pattern provides critical guidance for intervention optimization, where predominance of psychological benefits pathways (51.2 %) indicates interventions should prioritize subjective experience enhancement over objective exercise parameters, challenging traditional exercise prescription models emphasizing duration, intensity, and frequency metrics while potentially overlooking psychological contexts (Huang et al., 2023;Lorente et al., 2025).

### 5.3 Theoretical implications and preventive medicine applications

The findings advance theoretical understanding by demonstrating that exercise-emotion relationships in adolescence operate through temporally-ordered, cascading adaptation processes rather than simple direct mechanisms. The sequential mediation framework provides sophisticated conceptualization of how exercise benefits unfold over time, with implications for understanding developmental timing and sequencing of psychological adaptations. The substantial psychological benefits pathway positions positive affect not merely as exercise outcomes, but as mechanisms enabling further psychological development through cognitive and social pathways.

For preventive medicine practice, the sequential mediation findings provide evidence-based guidance for developing exercise interventions targeting adolescent emotional regulation. The predominance of psychological benefits as mediating mechanisms indicates preventive interventions should emphasize subjective experience optimization through personalized activity selection, intrinsic motivation enhancement, and meaning-making facilitation around exercise experiences. The sequential social self-efficacy pathway provides guidance for incorporating social components, suggesting staged approaches wherein individual positive associations are established before introducing collaborative elements, optimizing both pathways while minimizing social performance anxiety that could undermine initial psychological benefit development.

### 5.4 Limitations and future directions

The cross-sectional design limits causal inferences regarding temporal sequencing of psychological adaptations, despite theoretical support for proposed sequences. Longitudinal investigations with multiple assessment points are essential for confirming developmental trajectories of sequential mediation processes. The substantial effect sizes may reflect cultural factors specific to Chinese adolescent populations, requiring cross-cultural validation for establishing generalizability across diverse populations. Future research should investigate temporal dynamics through ecological momentary assessment approaches capturing real-time relationships between exercise experiences, psychological benefits, social self-efficacy fluctuations, and emotional states. Advanced methodological approaches, including neuroimaging studies examining how exercise-induced neural changes correspond to psychological adaptation processes, would provide convergent validity while elucidating neurobiological substrates of psychological benefits and social self-efficacy development.

## 6 Conclusions

This investigation demonstrates that physical exercise influences adolescent negative emotions through sequential psychological adaptation mechanisms operating alongside direct neurobiological pathways, with effect sizes indicating substantial clinical significance for preventive medicine applications. The partial mediation pattern reveals exercise benefits emerge through multiple, complementary mechanisms, with psychological benefits serving as primary mediators and social self-efficacy development providing additional therapeutic value through sequential adaptation processes. The findings provide robust empirical support for integrated theoretical models incorporating both psychological and neurobiological mechanisms within developmentally-informed frameworks. From preventive medicine perspectives, the sequential mediation framework offers evidence-based guidance for intervention development prioritizing psychological experience optimization while maintaining sufficient exercise parameters to activate direct neurobiological benefits, with temporal sequencing providing roadmaps for staged intervention approaches maximizing both immediate and cascading therapeutic effects.

## CRediT authorship contribution statement

**Xin Yang:** Writing – review & editing, Writing – original draft, Visualization, Project administration, Methodology, Investigation, Funding acquisition, Formal analysis, Data curation, Conceptualization. **Liuheng Lu:** Writing – review & editing, Writing – original draft, Validation, Project administration, Methodology, Funding acquisition, Data curation, Conceptualization.

## Funding

This research was funded by (1) Health and Emergency Skills Training Center of Guangxi(grant number NO.HESTCG202304). (2) Self-financed Scientific Research Topics of Health Commission of Guangxi Zhuang Autonomous Region(No.:*Z*-A20220394).

## Declaration of competing interest

The authors declare that they have no known competing financial interests or personal relationships that could have appeared to influence the work reported in this paper.

## Data Availability

Data will be made available on request.
